# Real-World Experience with Cangrelor as Adjuvant to Percutaneous Coronary Intervention: A Single-Centre Observational Study

**DOI:** 10.1155/2023/3197512

**Published:** 2023-06-16

**Authors:** Troels Thim, Lars Jakobsen, Rebekka Vibjerg Jensen, Nicolaj Støttrup, Ashkan Eftekhari, Erik Lerkevang Grove, Sanne Bøjet Larsen, Jacob Thorsted Sørensen, Steen Carstensen, Sahar Amiri, Karsten Tange Veien, Evald Høj Christiansen, Christian Juhl Terkelsen, Michael Maeng, Steen Dalby Kristensen

**Affiliations:** ^1^Department of Cardiology, Aarhus University Hospital, Aarhus, Denmark; ^2^Department of Clinical Medicine, Faculty of Health, Aarhus University, Aarhus, Denmark; ^3^Department of Cardiology, Aalborg University Hospital, Aalborg, Denmark; ^4^Emergency Department, Bispebjerg University Hospital, Bispebjerg, Denmark

## Abstract

**Background:**

Reversible P2Y12 inhibition can be obtained with cangrelor administered intravenously. More experience with cangrelor use in acute PCI with unknown bleeding risk is needed.

**Objectives:**

To describe real-world use of cangrelor including patient and procedure characteristics and patient outcomes.

**Methods:**

We performed a single-centre, retrospective, and observational study including all patients treated with cangrelor in relation to percutaneous coronary intervention at Aarhus University Hospital during the years 2016, 2017, and 2018. We recorded procedure indication and priority, the indications for cangrelor use, and patient outcomes within the first 48 hours after initiation of cangrelor treatment.

**Results:**

We treated 991 patients with cangrelor in the study period. Of these, 869 (87.7%) had an acute procedure priority. Among acute procedures, patients were mainly treated for STEMI (*n* = 723) and the remaining were treated for cardiac arrest and acute heart failure. Use of oral P2Y12 inhibitors prior to percutaneous coronary intervention was rare. Fatal bleeding events (*n* = 6) were only observed among patients undergoing acute procedures. Stent thrombosis was observed in two patients receiving acute treatment for STEMI. Thus, cangrelor can be used in relation to PCI under acute circumstances with advantages in terms of clinical management. The benefits and risks, in terms of patient outcomes, should ideally be assessed in randomized trials.

## 1. Introduction

Cangrelor is a reversible P2Y12 inhibitor administered intravenously with rapid onset and offset of action [1]. The use of cangrelor in relation to percutaneous coronary intervention (PCI) was introduced in the CHAMPION studies [2–4]. Offering reversible P2Y12 inhibition with rapid onset and offset, cangrelor seems attractive in the treatment of acutely ill patients undergoing PCI, in particular if the patients are not treated with oral P2Y12 inhibitors or if the risk of bleeding is uncertain. Such patients were not included in the CHAMPION studies, and randomized clinical trials including such patients are difficult to perform. Still, cangrelor is used clinically under these circumstances, and only limited registry data regarding this use have been reported [5]. More experience with cangrelor use in acute PCI with unknown bleeding risk should be gathered and shared.

We aimed to evaluate the benefit and risk of cangrelor and performed individual patient file review among all patients treated with cangrelor in relation to PCI at our institution during the years 2016–2018.

## 2. Methods

This study was a single-centre, retrospective, and observational study. Ethical committee approval was not required according to national and institutional guidelines.

### 2.1. Setting and Participants

We included all patients treated with cangrelor in relation to PCI at Aarhus University Hospital during the years 2016, 2017, and 2018. Cangrelor was introduced in Denmark during the year 2016.

The electronic patient file systems at our institution allowed identification of all patients treated with cangrelor. There was no control group. If a patient had received cangrelor treatment on more than one occasion in the study period, only the first treatment was used in this report. The follow-up period was 48 hours after cangrelor administration as in the CHAMPION studies [2–4].

The standard administration of cangrelor consists of an intravenous bolus dose of 30 *μ*g/kg followed by a 4 *μ*g/kg/min intravenous infusion for two hours.

For patients transitioning to oral ticagrelor, the ticagrelor bolus dose (180 mg) was administered as early as possible during cangrelor infusion. For patients transitioning to oral clopidogrel, the clopidogrel bolus dose (600 mg) was administered during the last half hour of the duration of the cangrelor infusion.

In patients where percutaneous haemodynamic support, such as VA-ECMO or Impella, is established, we usually decrease from 4 *μ*g/kg/min intravenous infusion after two hours to 0.75 *μ*g/kg/min intravenous infusion until removal of the percutaneous haemodynamic support system and then transition to oral P2Y12 inhibition as described above [6].

### 2.2. Variables

We retrieved all data from the electronic patient file systems through individual patient file review and from the Western Denmark Heart Registry in which all PCIs at our institution are registered in detail [7]. Study data were collected and managed using REDCap hosted at Aarhus University.

The outcomes were evaluated by the authors based on the information in the electronic patient file systems. Bleeding outcomes were registered in agreement with the Bleeding Academic Research Consortium (BARC) definitions [8]. We did not include type 1 and 2 bleeding. Also, type 4 (coronary artery bypass surgery-related bleeding) was not included. Thus, we registered type 3 and 5 bleeding events only. Bleeding events were hierarchical, i.e., patients with type 5 bleeding were not recorded as having type 3 bleeding and bleeding type 3b and 3c excluded bleeding type 3a. Cardiac death was death not caused by bleeding or any other overt non-cardiac cause. Acute myocardial infarction and stent thrombosis were registered according to the Fourth Universal Definition of Myocardial Infarction and Academic Research Consortium definitions [9, 10].

## 3. Result

### 3.1. Setting and Participants

We identified 991 patients treated with cangrelor in relation to PCI during the study period. The first patient was treated in July 2016, and the last patient in the cohort was treated in December 2018. In this period, a total of 7022 PCIs were performed at our institution. Of these, 2028 (29%) were acute, 2280 (32%) were subacute, and 2714 (39%) were elective.

**Figure 1:**
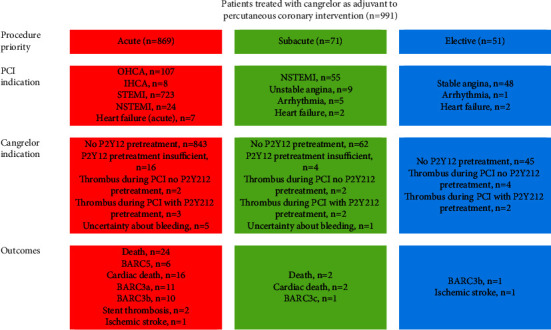
Outcomes and their relation to procedure priority and indications for percutaneous coronary intervention and cangrelor.

Characteristics of the 991 patients treated with cangrelor are reported in Table 1. The procedural characteristics are reported in Table 2. The indication for cangrelor use and concomitant antithrombotic treatment is reported in Table 3.  Figure 1 combines data from Tables 2 and 3 to illustrate the relation between procedure priority and indications for PCI and cangrelor and outcomes.  Figure 1 also includes data from Table 4 to illustrate the relation to outcomes. The majority of procedures were acute, and the majority of patients did not receive P2Y12 inhibitors prior to the procedure.

Therapeutic hypothermia after cardiac arrest was used in 80 (8.1%) patients receiving cangrelor. Femoral access for PCI was used more commonly than in our average practice.

## 4. Outcomes

Clinical outcomes are presented in Table 4. All six patients with fatal bleeding (BARC 5a and BARC 5b) were patients undergoing acute procedures. Four of the fatal bleeding events occurred in patients resuscitated after out-of-hospital cardiac arrest, and two had severe intra-abdominal haemorrhage and one had severe intrathoracic bleeding. These events were presumed to be complications to cardiopulmonary resuscitation including chest compressions. One patient had collapsed with cardiac arrest and suffered cranial trauma with intracranial bleeding. The last two fatal bleeding events were in patients presenting late with acute myocardial infarction of which one had pericardial tamponade within hours after PCI, while the other had low ejection fraction, preexisting anemia, and probable severe retroperitoneal haemorrhage (BARC 5a).

Among the eleven patients with BARC 3a, two had gastrointestinal bleeding, two had pleural haemorrhage after cardiopulmonary resuscitation, and one had femoral access site haematoma. The remaining six patients were treated with VA-ECMO (*n* = 4) or Impella (*n* = 2) and had different potential bleeding causes and need for transfusion. Among the eleven patients with BARC 3b, two had pleural haemorrhage after cardiopulmonary resuscitation and two had pericardial bleeding due to coronary perforations as complication to primary PCI. The remaining six patients were treated with VA-ECMO (*n* = 5) or Impella (*n* = 1) and had different potential bleeding causes and need for transfusion. One patient with BARC 3c had intracerebral haemorrhage diagnosed after PCI for presumed NSTEMI. In retrospect, this intracerebral haemorrhage may have been the primary cause for admission with general discomfort, nausea, vomiting, and mildly elevated cardiac biomarkers.

Among the 10 patients with prior intracranial bleeding, we recorded no bleeding events. Among the 29 patients with prior ischemic stroke or TIA, we recorded two patients with BARC 3a bleeding.

Among the 26 patients dying within 48 hours after cangrelor administration, the cause of death was bleeding in six patients as described above while two suffered fatal hypoxic brain injury after cardiac arrest. The remaining 18 deaths were considered of cardiac causes such as severe acute heart failure, arrhythmia, or ventricular septal defects.

Two patients treated for STEMI developed myocardial infarction caused by stent thrombosis in the newly implanted stent. In both cases, there was considerable thrombus in the right coronary artery initially and this was the reason for using cangrelor. In both cases, cangrelor was combined with oral loading with ticagrelor during the procedure and oral maintenance therapy with ticagrelor after the procedure. In one case, stent thrombosis occurred one hour after cangrelor bolus and two-hour cangrelor infusion had been completed. In the other case, cangrelor bolus and infusion were also combined with a 12-hour infusion of abciximab and stent thrombosis occurred 10 hours after abciximab infusion, i.e., 20 hours after cangrelor infusion, had been completed.

Two patients were considered to have stroke. One patient treated for STEMI developed weakness in the left arm which almost completely resolved within two days while one patient treated for stable angina developed light visual impairment on one eye and was diagnosed with a retinal artery thrombosis.

## 5. Discussion

This study describes the use of cangrelor in a large tertiary university hospital where primary PCI has been performed 24/7 for more than 25 years. In accordance with previous reports, the patients receiving cangrelor treatment at our institution are more acutely ill than the patients treated in the CHAMPION studies [2–4]. From the SCAAR, the results from 899 STEMI patients treated with primary PCI have been reported [5].

Our rationale for using cangrelor in the patients undergoing PCI is primarily based on the safety observed in the CHAMPION studies and the pharmacological properties offering rapid-onset effective P2Y12 inhibition and the possibility for rapid offset if needed in the acute setting [1].

The strength of this study is that it reports the outcomes of the patients treated with cangrelor in real-world clinical practice. Similar to previous reports [5], the major limitation is the lack of a control group to which outcomes can be compared. Without such a comparison, the true benefits and risks of cangrelor treatment in these critically ill patients are difficult to estimate. The study population mainly consisted of patients with acute coronary syndrome, and these patients have high risks of both ischemic events and bleeding [11]. Balancing of these risks to optimize patient outcomes remains the clinical task.

According to the ESC 2017 STEMI Guidelines, cangrelor may be considered in patients not pretreated with oral P2Y12 receptor inhibitors at the time of primary PCI (IIb level of evidence A), whereas GP IIb/IIIa inhibitors should be considered as bailout therapy in the event of angiographic evidence of large thrombus, slow or no reflow, and other thrombotic complications (IIa level of evidence C) [12]. In many centres including our own, cangrelor is now first choice of intravenous platelet inhibition in complex primary PCI. We need more studies to compare GP IIb/IIIa inhibitors with cangrelor in these patients.

In terms of practical clinical management, however, there are, in our experience, advantages with cangrelor treatment. In our catchment area, patients with STEMI and selected patients with cardiac arrest are admitted directly to the catheterization laboratory. These patients do not receive oral P2Y12 inhibitors en route. If acute angiography is followed by ad hoc PCI, cangrelor can be administered for immediate and effective P2Y12 inhibition without concerns of delayed gastric absorption, vomiting, or inability to take tablets due to depressed consciousness. In some instances, cardiopulmonary resuscitation has been administered and potential complications hereto, such as trauma to the liver, spleen, or lungs, are uncertain and difficult to diagnose prior to emergency PCI. In these instances, cangrelor can be administered until a decision regarding oral P2Y12 inhibitor initiation can be made based on further assessment, e.g., with a CT scan of the head, thorax, abdomen, and pelvis. In cases where clinically significant bleeding is observed, cessation of cangrelor and thereby fast termination of P2Y12 inhibition can be used in combination with thorough observation, conservative management, or surgery. When haemodynamic support such as VA-ECMO or Impella is used, extended duration of cangrelor infusion with a lower infusion dose can be used until after weaning from haemodynamic support [6]. Hereby, the shift to oral P2Y12 inhibition can be postponed until access sites have been safely closed either percutaneously or surgically.

## 6. Conclusion

Cangrelor can be used in relation to acute PCI with advantages in clinical management. In terms of patient outcomes, the benefits and risks of cangrelor should ideally be assessed in randomized clinical trials.

## Figures and Tables

**Table 1:** Patient characteristics (*n* = 991).

Age (years)	65.8 (56.3–74.9) [26.7–98.7]
Sex	
Female	244 (24.6%)
Male	747 (75.4%)
Hypertension	430 (43.4%)
Hypercholesterolemia	308 (31.1%)
Diabetes mellitus	144 (14.5%)
Prior myocardial infarction	98 (9.9%)
Prior PCI	132 (13.3%)
Prior CABG	50 (5.0%)
Prior ischemic stroke or TIA	40 (4.0%)
Prior intracranial bleeding	10 (1.0%)
LVEF (%)	47 (40–55) [5–70]
Plasma creatinine (*μ*mol/l)	75 (63–92) [31–787]
Blood haemoglobin (mmol/l)	8.3 (7.7–9.0) [3.4–13.0]
Platelet count (10^9^/l)	235 (199–278) [43–723]

PCI: percutaneous coronary intervention; CABG: coronary artery bypass grafting; TIA: transient ischemic attack; LVEF: left ventricular ejection fraction; eGFR: estimated glomerular filtration rate. Age, LVEF, plasma creatinine, blood haemoglobin, and blood thrombocytes are reported as median (quartiles) [range]. Other variables are reported as count (percent).

**Table 2:** PCI procedure characteristics (*n* = 991).

PCI procedure priority	
Acute	869 (87.7%)
Subacute	71 (7.2%)
Elective	51 (5.1%)
PCI procedure indication	
OHCA	107 (10.8%)
IHCA	8 (0.8%)
STEMI	723 (73.0%)
NSTEMI	79 (8.0%)
UAP	9 (0.9%)
SAP	48 (4.8%)
Arrhythmia	6 (0.6%)
Heart failure	11 (1.1%)
Access site for PCI	
Radial	520 (52.5%)
Femoral	467 (47.1%)
Brachial	4 (0.4%)
Mechanical ventilation during procedure	118 (11.9%)
Vasopressors and/or inotropes during procedure	109 (11.0%)
Haemodynamic support in during procedure	37 (3.7%)
Impella	19 (1.9%)
VA-ECMO	17 (1.7%)
IABP	1 (0.1%)
Aspiration thrombectomy	70 (7.1%)
Intervention	
Drug-eluting stent	943 (95.2%)
POBA (including DEB)	42 (4.2%)
No PCI (unsuccessful)	6 (0.6%)
Number of stents	
0	48 (4.8%)
1	483 (48.7%)
2	241 (24.3%)
3 or more	219 (22.1%)

PCI: percutaneous coronary intervention; OHCA: out-of-hospital cardiac arrest; IHCA: in-hospital cardiac arrest; STEMI: ST-segment elevation myocardial infarction; NSTEMI: non-ST-segment elevation myocardial infarction; UAP: unstable angina pectoris; VA-ECMO: venoarterial extracorporeal membrane oxygenation; IABP: intra-aortic balloon pump.

**Table 3:** Platelet inhibition and anticoagulation (*n* = 991).

Indication for cangrelor	
No pretreatment with oral P2Y12 inhibitor	950 (95.9%)
Pretreatment with oral P2Y12 inhibitor deemed insufficient	20 (2.0%)
Thrombus evolves during PCI without pretreatment with oral P2Y12 inhibitor	8 (0.8%)
Thrombus evolves during PCI despite pretreatment with oral P2Y12 inhibitor	7 (0.7%)
Uncertainty about bleeding and need for P2Y12 inhibition	6 (0.6%)
Aspirin prior to PCI	949 (95.8%)
Warfarin prior to PCI	17 (1.7%)
NOAC prior to PCI	27 (2.7%)
Oral P2Y12 inhibitor after PCI	
No	15 (1.5%)
Clopidogrel	95 (9.6%)
Ticagrelor	881 (88.9%)
Unfractionated heparin as adjunct to PCI	974 (98.3%)
Abciximab as adjunct to PCI	21 (2.1%)
Bivalirudin as adjunct to PCI	2 (0.2%)
Eptifibatide as adjunct to PCI	1 (0.1%)

PCI: percutaneous coronary intervention; NOAC: novel oral anticoagulants.

**Table 4:** Outcomes within 48 hours after cangrelor administration.

All-cause death	26 (2.6%)
BARC 5a	1 (0.1%)
BARC 5b	5 (0.5%)
Cardiac death	18 (1.8%)
Death, other	2 (0.2%)
Non-fatal bleeding more than BARC 2	
BARC 3a	11 (1.1%)
BARC 3b	11 (1.1%)
BARC 3c	1 (0.1%)
Acute myocardial infarction	2 (0.2%)
Stent thrombosis	2 (0.2%)
Ischemic stroke	2 (0.2%)

BARC: Bleeding Academic Research Consortium.

## Data Availability

The data are not publicly available due to Danish data protection regulations.
